# Research on the prevention and treatment of diabetes in the elderly through the integration of community service centers and exercise intervention

**DOI:** 10.1097/MD.0000000000047165

**Published:** 2026-01-16

**Authors:** Dan Shen, Yanyan Zhang, Xi’an Ni, Yongtao Shen, Liuliu Qian, Chunhui Li, Chunxia Sha

**Affiliations:** a Department of Scientific Research and Education, The Third People’s Hospital of Qidong, Qidong, Jiangsu Province, China; b Department of Public Health, The Third People’s Hospital of Qidong, Qidong, Jiangsu Province, China.

**Keywords:** diabetes, elderly chronic diseases, exercise intervention, health management

## Abstract

This study aims to investigate the effects of a three-month personalized exercise intervention on the physical, psychological, and metabolic outcomes of elderly individuals with diabetes in the Qidong community. The ultimate goal is to provide evidence for developing strategies and measures to prevent and treat geriatric diabetes and its complications in primary hospitals within the specific social context of urban communities. A single-group pre–post intervention study without a control group was conducted among 105 elderly patients with diabetes at the Physical Examination Center of Qidong Third People’s Hospital from 2023 to 2024. The intervention lasted for 3 months and included weekly personalized exercise sessions combined with wearable smart monitoring devices and 3 times weekly self-exercise feedback via smartphones. Changes in psychological status, physical functions (including body mass index [BMI], waist–hip ratio, body fat percentage, and blood pressure), and metabolic indicators (such as fasting blood glucose, blood lipids, and glycated hemoglobin) were assessed before and after the intervention using paired *t*-tests. After the three-month intervention, significant improvements were observed in the physical, psychological, and social functions of elderly diabetic patients (*P* <.05). Specifically, body weight decreased by 4.95 kg, BMI decreased by 1.68 kg/m^2^, and body fat percentage decreased by 1.65%, while lung capacity increased significantly (*P* <.05). No significant changes were observed in heart rate, while diastolic blood pressure, waist circumference, and hip circumference showed mild downward trends without statistical significance (*P* >.05). After the intervention, fasting blood glucose decreased by 1.22 mmol/L, glycosylated hemoglobin, type A1C decreased by 0.92%, and triglycerides decreased by 0.32 mmol/L, whereas serum high-density lipoprotein increased by 0.23 mmol/L (all *P* <.05). Total cholesterol and low-density lipoprotein showed slight but non-significant reductions (*P* >.05). Through the implementation of a structured exercise intervention program supported by general practitioners and specialized medical staff, primary hospitals partially improved exercise capacity and scientific health management among elderly patients with chronic diseases. These findings preliminarily demonstrate the potential benefits of integrating exercise intervention into community-based diabetes management and may provide a reference for the future development of integrated medical and preventive care models for geriatric chronic diseases.

## 1. Introduction

The elderly population in China, which is aged 60 and above, has reached 264 million according to the data from Seventh National Population Census, accounting for 18.7% of the total population.^[1]^ Along with the aging population, the incidence and prevalence of chronic non-communicable diseases such as hypertension, diabetes, and chronic obstructive pulmonary disease have generally shown an upward trend, with their complications becoming the leading causes of death among the elderly in China. Epidemiological surveys on diabetes show that the number of diabetic patients in China is approximately 113 million, accounting for 24% of the global total.^[2]^ Furthermore, multiple studies have found that the diabetes incidence rate in the population aged 65 and above in China is continuously increasing. Currently, the prevalence of diabetes among the elderly is 18.8%, accounting for 25% of the global elderly diabetic patients.^[3]^ Other research has indicated that the prevalence of diabetes in China is closely related to the rapid urbanization, population aging, obesity or overweight, and susceptibility genes.^[4]^ Due to the high incidence, long disease course, and low effective control rate of chronic diseases, these conditions not only impose a heavy burden on the country and families but also lead to a series of social problems.

In recent years, to tackle the challenges posed by an aging population and the increasing prevalence of chronic diseases, Chinese government has released several key documents. The “Healthy China 2030 Plan outlines the goal to “build an integrated healthcare service system,”^[5]^ while the “14th Five-Year Plan for National Health” emphasizes the goal of integrating medical prevention and treatment, focusing on “active prevention before illness, scientific management after illness, and continuous follow-up services” to form an integrated health management system^[6]^ With economic and social development and changes in the health and medical environment, elderly chronic disease patients have increasingly focused on and sought a high-quality life, which requires health management to evolve from a focus on chronic disease as a specialized medical perspective to a multi-dimensional, comprehensive health management approach incorporating medical knowledge, social environment, personal development, and artificial intelligence. This shift expands the scope and content of health management services. Research has confirmed that a lack of physical activity is a major cause of various diseases and health issues.^[7]^ Introducing physical activity interventions in the standardized management of chronic diseases and the prevention of high-risk groups can effectively control risk factors such as hypertension, diabetes, dyslipidemia, and obesity, thereby improving the overall management of chronic diseases, reducing the occurrence and development of these conditions, enhancing the physical fitness of chronic disease patients and high-risk groups, improving quality of life, and further reducing the disease burden.

Although numerous studies have confirmed the benefits of exercise in improving glycemic control and cardiovascular health in diabetic patients, there remains a lack of evidence regarding how exercise interventions can be systematically integrated into community-based primary healthcare models in China, especially for elderly individuals. Moreover, the application of smart wearable technologies for real-time monitoring and adherence enhancement in local hospital settings is still limited. Therefore, this study seeks to fill this gap by developing and evaluating a community-integrated, technology-assisted exercise intervention model implemented in a county-level hospital in Qidong.

This study aimed to evaluate the effects of a three-month personalized exercise intervention on measurable outcomes among elderly individuals with diabetes in the Qidong community. Based on an individualized health management plan combining exercise intervention, health education, blood glucose monitoring, and follow-up management, we assessed changes in metabolic indicators (fasting blood glucose, glycated hemoglobin, and blood lipids), physical parameters (body mass index [BMI], body fat percentage, blood pressure, and vital capacity), and psychosocial function (quality-of-life domains) before and after the intervention. We hypothesized that integrating personalized exercise programs into community-based primary healthcare, supported by smart wearable monitoring, would significantly improve the physical, metabolic, and psychosocial health of elderly diabetic patients and provide practical evidence for chronic disease management in community settings.

## 2. Materials and methods

### 2.1. Data collection

This single-group pre–post intervention study was conducted at the Chronic Disease Exercise Health Management Center of the Third People’s Hospital of Qidong City between 2023 and 2024. During this period, 35,869 elderly individuals participated in routine health checkups. Medical staff reviewed their examination reports and identified those with diabetes or at risk of diabetes. Eligible elderly individuals were invited to participate in the diabetes health management program involving a three-month exercise intervention.

All participants were screened according to predefined inclusion and exclusion criteria. Inclusion criteria were: Diagnosed with type 2 diabetes mellitus (T2DM) according to the Chinese Diabetes Society Guidelines (2024 edition); Aged ≥ 46 years, with stable disease status and no acute complications in the past 3 months; Medically cleared for moderate-intensity exercise (50–70% HRmax); Able to perform daily activities independently and use a smartphone for exercise feedback; and Provided written informed consent and willing to complete the three-month intervention.

Exclusion criteria were: Type 1 or secondary diabetes; Severe cardiovascular, hepatic, renal, or cerebrovascular diseases; Cognitive impairment, severe mental illness, or communication barriers; Musculoskeletal or neurological disorders that limit exercise; Long-term use of medications significantly affecting metabolism (e.g., corticosteroids); and Participation in other structured exercise or rehabilitation programs during the study period.

**Figure 1.**
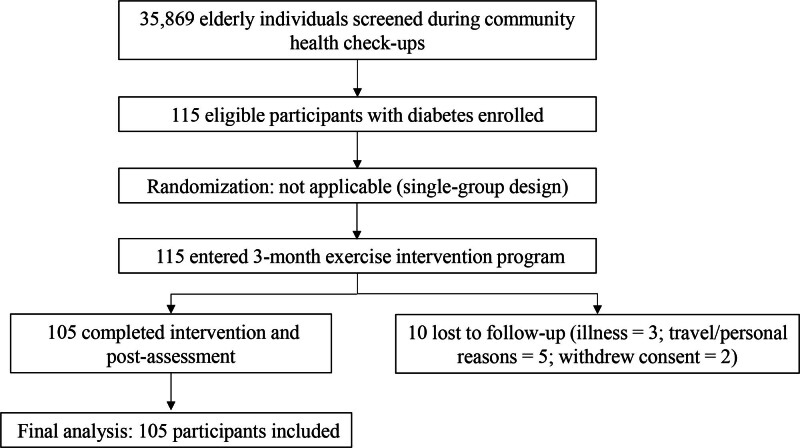
Flow diagram.

After screening, 115 elderly individuals met the eligibility criteria and were enrolled in the program. Written informed consent was obtained from all participants prior to inclusion. The study protocol was reviewed and approved by the Clinical Research Ethics Committee of the Third People’s Hospital of Qidong City (Ethical Approval No. QDSY-kyjyk-202311005). A flow diagram summarizing participant recruitment, inclusion, and follow-up is presented in Figure 1.

### 2.2. Methods

**Figure 2.**
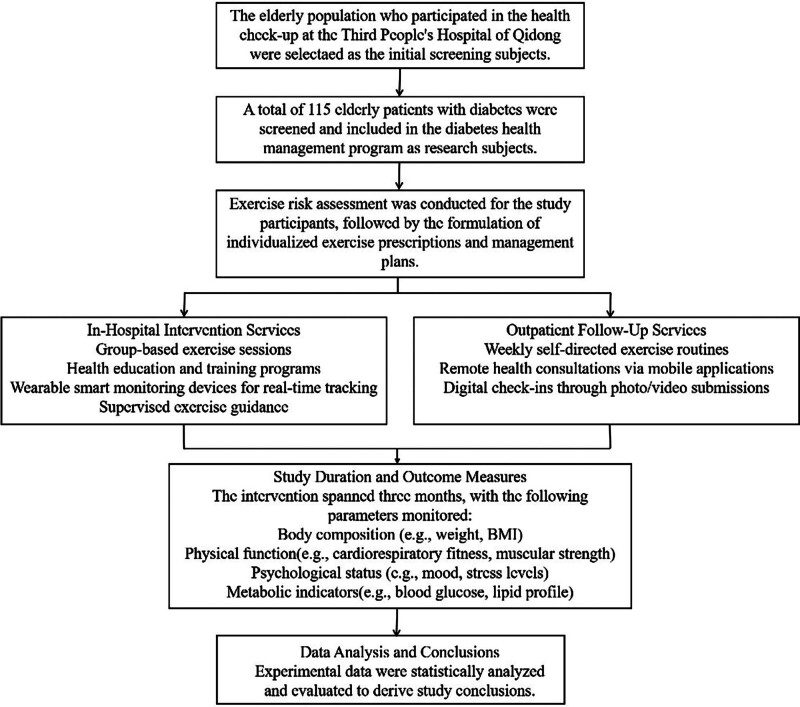
Hospital diabetes health management exercise intervention process.

Following the pre–post intervention design, our hospital formed an exercise intervention research team consisting of one exercise prescription specialist, 1 national-level sports coach, as well as several general practitioners and nurses. This team provided a 3-month personalized diabetes exercise health management program for elderly patients with chronic diseases, as shown in Figure 2.

Based on the study protocol, 115 elderly individuals with diabetes who participated in health checkups at the Third People’s Hospital of Qidong City were screened and recruited into the program under the principle of informed consent. A comprehensive exercise risk assessment was conducted for each participant using clinical examination results and physical function indicators. Individualized exercise prescriptions and management plans were then developed accordingly.

The intervention consisted of 2 complementary components: In-hospital intervention services, including group-based exercise sessions, health education and training programs, real-time monitoring using wearable smart devices, and supervised exercise guidance provided by professional staff; Outpatient follow-up services, involving weekly self-directed exercise routines, remote consultations via WeChat and other mobile applications, and digital check-ins through the submission of photos and videos.

Throughout the 3-month intervention period, participants’ body composition, physical function, psychological state, and metabolic indicators were continuously monitored. Upon completion, all experimental data were statistically analyzed and evaluated to derive the study conclusions.

### 2.3. Exercise intervention

1. Pre-exercise preparation: Laboratory tests were conducted to collect data on complete blood count, liver and kidney function, blood lipids, blood glucose, and glycated hemoglobin. Body composition measurements included data on body composition, cardiopulmonary function, muscle strength, etc. Questionnaire surveys were used to gather information such as the Diabetes Quality of Life Instrument (QLICD-DM V2.0) and exercise risk assessment questionnaires.
2. Personalized exercise prescription and adherence monitoring: Based on the assessment results, exercise prescription specialists designed personalized training plans in conjunction with smart wearable devices. The intervention lasted for 3 months, with participants exercising 3 to 5 times per week for 40 to 60 minutes per session. Exercise intensity was controlled at 60 to 70% of the age-predicted maximum heart rate (HRmax) or approximately 4 to 6 metabolic equivalents (METs), corresponding to moderate intensity. Each session included aerobic, resistance, and flexibility training components. Aerobic activities involved brisk walking, treadmill exercise, cycling, and Baduanjin (a traditional Chinese exercise); resistance training included elastic-band or bodyweight exercises targeting major muscle groups; and flexibility training consisted of stretching during warm-up and cool-down (about 10 minutes each). Exercise intensity and adherence were continuously monitored by smart devices, which recorded heart rate, step count, and energy expenditure. Participants were also required to upload post-exercise logs through smartphone applications, including perceived exertion, exercise duration, and self-rated fatigue. Device data were synchronized weekly through the hospital’s health management platform and automatically cross-checked with self-reported logs. Participants were considered adherent if they completed at least 80% of the prescribed sessions within the target heart rate range. Any discrepancies between self-reports and device data were reviewed by exercise specialists and verified through telephone follow-ups. This combined objective–subjective monitoring approach enhanced the accuracy and reliability of adherence evaluation throughout the intervention.

### 2.4. Statistical analysis

Health checkup data were exported from the hospital’s checkup system and analyzed using SPSS 21.0 statistical software. Normality of continuous variables was tested using the Shapiro–Wilk test before further analysis. Descriptive statistics for measurement data were expressed as mean ± standard deviation (mean ± SD), including age, basic physical data, metabolic indicators, etc. For variables not following a normal distribution, the Wilcoxon signed-rank test was applied. Paired *t*-tests were used to analyze the pre- and post-intervention changes in morphological indicators, blood glucose, and blood pressure. Given the exploratory nature of this study and the moderate number of comparisons, no formal adjustment for multiple testing was applied; however, all *P*-values were interpreted with caution. Of the 115 participants initially enrolled, 105 completed the 3-month intervention and post-assessment, whereas 10 participants (8.7%) were lost to follow-up and excluded from the final per-protocol analysis. Because of the small number of missing cases (n = 10) and the exploratory design, no imputation of missing data was performed; missing data were handled by listwise deletion. A *P*-value of <.05 was considered statistically significant. This study adopted a single-group pre–post intervention design, and no formal a priori sample-size calculation was conducted due to its exploratory nature. The final sample of 115 participants was determined by the number of eligible individuals who met inclusion criteria and consented to participate. A post hoc power analysis based on the observed change in fasting blood glucose (pre: 8.78 ± 0.75 mmol/L; post: 7.56 ± 0.93 mmol/L; Δ = −1.22 mmol/L) at a two-tailed α = 0.05 yielded a statistical power of approximately 0.92 under the observed effect size (Cohen *d* ≈ 1.45), indicating that the sample size was sufficient to detect meaningful within-group effects. However, as this was a pilot study without a control group, future large-scale randomized controlled trials are warranted to verify these findings. Given the exploratory nature of this pilot study and the focus on overall intervention effects, *P* values were reported using conventional thresholds (*P* <.05 or *P* >.05) rather than exact values.

## 3. Results

### 3.1. Clinical data

A background survey was conducted before the exercise intervention to collect data on various factors such as gender, age, disease duration, education level, income, living situation, social support, and interests of the 115 study participants. The baseline demographic and clinical characteristics are summarized in Table 1, indicating that the sample was generally homogeneous and representative of the elderly diabetic population in the community.

**Table 1:** Basic information of elderly diabetic patients (n = 115).

Item	Male (31)	Female (84)
Age	58 ± 8.14	56 ± 10.06
Disease duration		
<5 yr	4 (12.90%)	33 (39.29%)
5–10 yr	20 (64.52%)	42 (50.00%)
>10 yr	7 (22.58%)	9 (10.71%)
Economic level		
<1500	3 (9.68%)	10 (11.90%)
1500–4000	22 (70.97%)	62 (73.81%)
>4000	6 (19.35%)	12 (14.29%)
Education level		
Primary school or below	5 (16.13%)	7 (8.33%)
Junior high and high school	22 (70.97%)	61 (72.62%)
College or above	4 (12.90%)	16 (19.05%)
Marital status		
Married/Co-habiting	28 (90.32%)	74 (88.10%)
Divorced/Widowed/Separated	3 (9.68%)	10 (10.90%)
Bad habits (smoking, drinking)		
Never	1 (3.22%)	72 (85.71%)
Occasionally	8 (25.81%)	12 (14.29%)
Frequently	22 (70.97%)	0 (0.00%)

Data are presented as n, mean ± SD, (%).

### 3.2. Comparison of physical results of elderly diabetic patients before and after exercise intervention

After 3 months of personalized exercise intervention, elderly diabetic patients showed a weight reduction of about 6 to 8%, waist circumference decreased by about 2 to 3%, and significant reductions in BMI and body fat percentage compared to pre-intervention levels (*P* <.05). These results indicate that the structured exercise program effectively improved body composition and reduced obesity-related risk factors in older adults. Detailed results are shown in Table 2.

**Table 2:** Comparison of physical indicators before and after exercise intervention in elderly diabetic patients (n = 105).

Basic indicators	Before exercise intervention	After exercise intervention
Weight (kg)	69.58 ± 11.71	64.63 ± 11.89*
BMI (kg/m^2^)	26.51 ± 3.90	24.83 ± 4.38**
Waist circumference (cm)	87.36 ± 13.89	85.40 ± 13.58
Hip circumference (cm)	102.58 ± 4.18	101.34 ± 4.31
Waist-hip ratio	0.85 ± 0.10	0.84 ± 0.09
Body fat percentage (%)	28.54 ± 4.02	26.89 ± 3.96*

Data are presented as mean ± SD. Paired *t*-test was used to compare pre- and post-intervention values. *P* < .05 indicates statistical significance.

BMI = body mass index.

* Indicates a statistically significant difference compared to before exercise intervention (*P* < .05).

** Indicates *P* < .01.

### 3.3. Comparison of physical and psychological function results before and after exercise intervention in elderly diabetic patients

After 3 months of personalized exercise intervention, elderly diabetic patients showed a significant decrease in systolic blood pressure and a notable increase in vital capacity compared to pre-intervention levels (*P* <.05). However, there were no significant changes in heart rate. Psychological function showed improvement, and while there was a trend of reduced diastolic blood pressure, the difference was not statistically significant. These findings demonstrate that regular, moderate-intensity exercise not only contributes to better cardiovascular stability and pulmonary function but also has positive psychosocial benefits for elderly patients. Detailed results are shown in Tables 3 and 4.

**Table 3:** Comparison of physical function indicators before and after exercise intervention in elderly diabetic patients (n = 105).

Physical function indicators	Before exercise intervention	After exercise intervention
Heart rate (beats/min)	75.20 ± 6.49	74.86 ± 8.36
Systolic blood pressure (mm Hg)	136.43 ± 15.32	129.84 ± 13.58*
Diastolic blood pressure (mm Hg)	85.26 ± 10.43	82.65 ± 9.78
Vital capacity (mL)	3258.74 ± 249.61	3379.81 ± 339.39**

Data are presented as mean ± SD. Paired *t*-test was used to compare pre- and post-intervention values. *P* < .05 indicates statistical significance.

* Indicates a statistically significant difference compared to before exercise intervention (*P* <.05);

** Indicates *P* <.01.

**Table 4:** Comparison of quality of life scores before and after exercise intervention in elderly diabetic patients (n = 105).

	Physical function	Psychological function	Social function
Before exercise intervention	66.48 ± 15.92	70.16 ± 22.95	68.55 ± 14.73
After exercise intervention	70.81 ± 18.33	82.51 ± 20.75*	77.12 ± 18.06*

Data are presented as mean ± SD. Paired *t*-test was used to compare pre- and post-intervention values. *P* <.05 indicates statistical significance.

* Indicates a significant difference compared with before exercise intervention (*P* <.05).

** Indicates *P* <.01.

### 3.4. Comparison of laboratory indicators in elderly diabetic patients before and after exercise intervention

After 3 months of personalized exercise intervention, elderly diabetic patients showed a decrease in fasting blood glucose by approximately 11 to 16%, glycosylated hemoglobin, type A1C (HbA1c) decreased by 10 to 12%, triglycerides decreased, and serum high-density lipoprotein increased, with statistical significance (*P* <.05). Total cholesterol and low-density lipoprotein showed a downward trend, but the difference was not statistically significant. These improvements in glycemic and lipid profiles indicate enhanced metabolic control and reduced cardiovascular risk following regular exercise. Details are presented in Table 5.

**Table 5:** Comparison of laboratory indicators in elderly diabetic patients before and after exercise intervention (n = 105).

Laboratory indicators	Before exercise intervention	After exercise intervention
Fasting blood glucose (mmol/L)	8.78 ± 0.75	7.56 ± 0.93**
HbA1c (%)	7.87 ± 1.43	6.95 ± 1.21*
Triglycerides (mmol/L)	1.64 ± 1.03	1.32 ± 0.98*
Total cholesterol (mmol/L)	4.96 ± 1.17	4.82 ± 1.21
High-density lipoprotein	1.21 ± 0.35	1.44 ± 0.28*
Low-density lipoprotein	2.74 ± 0.90	2.62 ± 0.68

Data are presented as mean ± SD. Paired *t*-test was used to compare pre- and post-intervention values. *P* <.05 indicates statistical significance.

HbA1c = glycosylated hemoglobin, type A1C.

* Indicates a significant difference compared with before exercise intervention (*P* <.05).

** Indicates *P* <.01.

**Figure 3.**
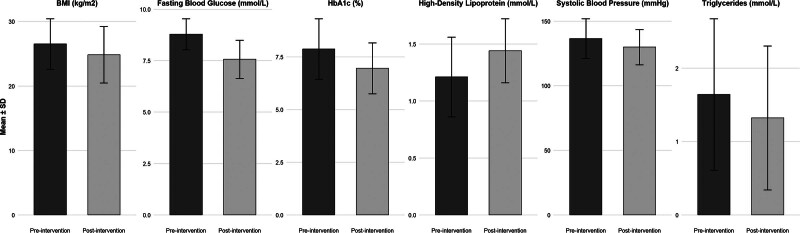
Changes in key physical and metabolic indicators before and after the 3-mo exercise intervention (n = 105).

As shown in Figure 3, the intervention led to consistent improvements across key physiological and metabolic parameters, including reductions in BMI, systolic blood pressure, fasting blood glucose, HbA1c, and triglycerides, together with an increase in HDL.

## 4. Discussion

Community-based primary health institutions play a crucial role in chronic disease prevention and management. In this pre–post intervention study, a structured exercise health management program was implemented for elderly individuals with diabetes through coordinated efforts between hospitals and community services. The program, involving personalized exercise prescriptions, health education, and digital monitoring, appeared to improve physical and psychological health outcomes among participants. These findings suggest that integrating medical supervision with community-based exercise management may be a feasible and beneficial approach for elderly diabetic populations.

Consistent with existing literature, we believe that factors such as gender, age, economic income, education level, and disease duration have an impact on chronic disease health management in elderly diabetic patients.^[8]^ Therefore, in the background survey of elderly diabetic patients, a targeted approach to health education on diabetes and exercise management is essential. This enables the elderly participants to form a correct understanding of diabetes. Additionally, the study project also enriches the leisure activities of the elderly, broadens their social circles, and better meets their social integration and mental health needs, thereby improving the quality of life for elderly residents in the community.

After 3 months of personalized exercise intervention, participants demonstrated moderate but meaningful improvements in several physical and functional indicators. Reductions were observed in body weight, waist circumference, BMI, and body fat percentage, accompanied by lower systolic blood pressure and enhanced pulmonary function. These findings suggest that a combination of aerobic and resistance exercises may be beneficial for elderly individuals with diabetes by improving body composition and cardiopulmonary performance. Similar results have been reported in previous studies, which indicated that regular aerobic exercise contributes to vascular remodeling and plays an important role in chronic disease management.^[9]^

With respect to metabolic outcomes, the intervention was associated with improved glycemic and lipid control, as reflected by decreases in fasting blood glucose, HbA1c, and triglycerides, along with an increase in serum high-density lipoprotein levels. Total cholesterol and low-density lipoprotein showed a downward trend, though not statistically significant. These findings are consistent with previous evidence that aerobic exercise enhances insulin sensitivity and glucose metabolism by modulating insulin transporter proteins, improving insulin resistance, and reducing oxidative stress.^[10,11]^ Moreover, exercise is known to play a key role in regulating lipid metabolism.^[12]^ The observed reduction in HbA1c likely reflects improved peripheral glucose uptake and more stable glycemic control induced by enhanced muscular insulin responsiveness. Similarly, the decrease in triglycerides is consistent with increased lipoprotein lipase activity and accelerated fatty acid oxidation during regular aerobic and resistance training.^[13,14]^ Together, these physiological adaptations support the role of structured exercise in improving overall metabolic homeostasis in elderly diabetic patients.

Currently, in China, there are various models for health management of elderly chronic diseases, most of which emphasize the integration of community-based general practitioner teams and self-management of chronic diseases. These models intervene in areas such as assessing the patient’s personal health status, developing personalized treatment plans, and evaluating treatment adherence.^[15]^ In this research project, we relied on government policies as guidance, with hospitals as the main entity, targeting elderly diabetic patients. We integrated health monitoring and assessment, sports medicine, disease treatment and prevention, and other related medical knowledge to preliminarily validate the effectiveness of the hospital health guidance center model within the context of the integration of medical and technological services. The integration of community service centers in this program was reflected in several aspects. Specifically, community physicians and general practitioners collaborated with hospital specialists to conduct health education, deliver individualized exercise prescriptions, and perform continuous follow-up and monitoring. Local community centers provided venues and basic exercise equipment for group training and periodic physical assessments, while community health staff assisted in organizing classes, collecting feedback, and maintaining regular communication with participants. Through this collaborative model, medical expertise, community engagement, and digital monitoring formed a closed-loop management system that enhanced accessibility, continuity, and sustainability of diabetes health management at the primary-care level.

This study does have some limitations. First, it mainly serves as an exploratory pilot study on exercise interventions for elderly diabetic patients in Qidong City. Due to time and funding constraints, the sample size was relatively small. Therefore, the conclusions should be applied to other elderly populations with chronic diseases based on specific circumstances. Second, considering that the study primarily targeted elderly diabetic patients in the community, data were mainly collected from patient health checkup results, medical records, and smart device monitoring. However, the influence of factors such as economic status, education level, lifestyle, and social conditions on the outcomes remains to be further explored. Third, some exercise-related variables, including duration, frequency, and intensity, were self-reported by participants, which may have introduced reporting bias. In addition, potential confounding factors such as dietary habits, medication adjustments, or seasonal variations were not fully controlled, which may have influenced metabolic outcomes. Most importantly, the absence of a control group limits the ability to infer causality, as the observed improvements might be partially influenced by uncontrolled external factors or natural fluctuations. Future large-scale randomized controlled trials are warranted to validate these preliminary findings and clarify causal mechanisms.

## 5. Conclusion

This study was conducted in response to practical healthcare needs, emphasizing social responsibility and the pursuit of public health benefits. It explored the feasibility of a community-based exercise intervention program for elderly diabetic patients, analyzed its implementation process, and summarized preliminary experience from hospital–community collaboration. By integrating primary healthcare services with professional exercise guidance and psychological support, the program appeared to promote improvements in physical condition, metabolic indicators, and mental well-being among elderly individuals with chronic diseases. Moreover, it may help foster healthier lifestyles and raise awareness of chronic disease prevention and management within the community. Although the findings provide early evidence supporting the potential benefits of this integrated model, they should be interpreted cautiously due to the absence of a control group and the limited sample size. Future large-scale, controlled studies are needed to further validate these results and refine sustainable strategies for community-based diabetes health management.

## Acknowledgments

We would like to express our sincere gratitude to colleagues from other departments for their valuable assistance throughout the research process, and to our department colleagues for their collaborative efforts during the manuscript writing. We also extend our appreciation to the Quality of Life and Applied Psychology Research Center at Guangdong Medical University for their support of this research project and for providing the diabetes survey questionnaire used in this study.

## Author contributions

**Conceptualization:** Dan Shen.

**Data curation:** Yanyan Zhang.

**Formal analysis:** Xi’an Ni.

**Funding acquisition:** Dan Shen.

**Investigation:** Yongtao Shen.

**Project administration:** Chunxia Sha.

**Resources:** Chunhui Li.

**Validation:** Xi’an Ni.

**Visualization:** Liuliu Qian.

**Writing – original draft:** Dan Shen.

**Writing – review & editing:** Dan Shen, Yanyan Zhang, Xi’an Ni, Yongtao Shen, Liuliu Qian, Chunhui Li, Chunxia Sha.
